# Deep-Learning with Domain-Specific Pretraining for Breast Cancer Neoadjuvant Chemotherapy Response Prediction from Pre-Treatment B-Mode Ultrasound

**DOI:** 10.3390/cancers18091345

**Published:** 2026-04-23

**Authors:** Christoph Fürböck, Ivana Janickova, Georg Langs, Thomas H. Helbich, Paola Clauser, Raoul Varga, Pascal Baltzer, Panagiotis Kapetas

**Affiliations:** 1 Department of Biomedical Imaging and Image-guided Therapy, Computational Imaging Research Lab, Medical University of Vienna, 1090 Vienna, Austria; christoph.fuerboeck@meduniwien.ac.at (C.F.); georg.langs@meduniwien.ac.at (G.L.); 2 Comprehensive Center for Artificial Intelligence in Medicine, Medical University of Vienna, 1090 Vienna, Austria; 3 Department of Biomedical Imaging and Image-guided Therapy, Christian Doppler Laboratory for Machine-Learning-Driven Precision Imaging, Medical University of Vienna, 1090 Vienna, Austria; 4 Department of Biomedical Imaging and Image-guided Therapy, Division of General and Pediatric Radiology, Medical University of Vienna, 1090 Vienna, Austria; paola.clauser@meduniwien.ac.at; 5 Department of Biomedical Imaging and Image-guided Therapy, Division of Cardiovascular and Interventional Radiology, Medical University of Vienna, 1090 Vienna, Austria; raoul.varga@meduniwien.ac.at; 6 Department of Biomedical Imaging and Image-guided Therapy, High Field Magnetic Resonance Center (HFMRC), Medical University of Vienna, 1090 Vienna, Austria; pascal.baltzer@meduniwien.ac.at; 7 Department of Radiology, Columbia University Irving Medical Center, Vagelos College of Physicians and Surgeons, New York, NY 10032, USA

**Keywords:** deep learning, breast cancer, ultrasound

## Abstract

**Objective:** We evaluated whether a deep-learning model could predict the response to neoadjuvant chemotherapy (NAC) in breast cancer using the pre-treatment B-mode ultrasound. **Methods:** This retrospective study included 245 female patients (253 lesions) treated with NAC between 2017 and 2019. Lesions were categorized as complete response (CR; 103) or non-CR (150) based on postoperative pathology. We trained ResNet18-based models using pre-treatment B-mode ultrasound images (Image) and clinical features. Three training strategies were evaluated: training from scratch (SC); transfer learning (TL); and domain-specific pretraining (USP). Predictive performance was assessed using descriptive statistics. **Results:** The best-performing model (USP Image) achieved 0.76 accuracy (specificity: 0.80; sensitivity: 0.72), significantly outperforming all other models, including those that used additional clinical features (p<0.05). USP improved performance across most model types compared to SC and TL, highlighting the value of domain-specific pretraining. Clinical features added value with SC or TL, but not with USP, suggesting that pretrained models can extract the most relevant information directly from images. Grad-CAM analysis revealed that non-CR predictions focused on the tumor and posterior shadowing—features linked to chemoresistant subtypes. CR predictions focused mainly on more heterogeneous, peritumoral regions. **Conclusions:** This finding underscores ultrasound’s potential as a low-cost, accessible tool for predictive oncology in personalized, AI-driven treatment planning.

## Simple Summary

In the era of personalized care, it is important to predict how a specific breast cancer will respond to chemotherapy, even before the initiation of therapy. In this study, we developed and evaluated a deep-learning model that uses pre-treatment B-mode ultrasound images to predict response to neoadjuvant chemotherapy. Our best-performing model achieved an accuracy of 76% in distinguishing tumors that demonstrated a pathological complete response (CR) from those that did not. Of various training approaches, the one that utilized ultrasound-specific pretraining achieved the best performance compared to other approaches, while the addition of clinical information did not further improve these results. Grad-CAM visual explanation maps showed that, in non-CR tumors, attention was mainly focused to the tumor and posterior shadowing, whereas in tumors showing CR more attention was shown to heterogeneous peritumoral regions. Our findings illustrate the potential of the combination of ultrasound with AI as a cost-effective, interpretable tool to support treatment planning in breast cancer.

## 1. Introduction

Breast cancer (BC) remains one of the most prevalent and challenging malignancies globally, with the highest incidence among all cancer types in females worldwide [1] and the highest mortality among the 10 most common cancer types in females [1]. Despite advancements in screening, diagnostic modalities, and treatment options, BC management remains complex due to its heterogeneity. Tumor biology, molecular subtypes, and individual patient characteristics all contribute to variability in disease progression and therapeutic response.

Neoadjuvant chemotherapy (NAC) is frequently employed in BC management for both locally advanced cases [2] and early-stage, biologically aggressive tumors, for which systemic treatment is indicated [3]. NAC can reduce tumor size to enable breast-conserving surgery. In addition, the response provides insight into tumor biology, and serves as an early indicator of systemic therapy efficacy [4]. Not all patients respond favorably to NAC, and unnecessary exposure to its toxic side effects can significantly affect the quality of life and long-term health [5,6]. Therefore, accurately predicting a patient’s response to NAC before initiating treatment is of critical clinical importance. A reliable predictive model could aid in treatment stratification, allowing non-responders to explore alternative therapies and improving overall outcomes.

Medical imaging has played a pivotal role in assessing treatment response. Magnetic resonance imaging (MRI) is considered the gold standard for the evaluation of breast tumors due to its high contrast resolution and ability to capture both functional and morphological information. MRI-based studies have shown promise in predicting NAC response using advanced radiomics and deep-learning techniques [7,8]. However, MRI has significant drawbacks, including high cost, limited accessibility, longer acquisition times, and contraindications for certain patient populations (e.g., those with implants or claustrophobia). These limitations underscore the need for alternative, cost-effective, and accessible imaging techniques.

Ultrasound (US) offers a compelling alternative. It is widely available, non-invasive, cost-effective, and does not involve ionizing radiation. In the context of BC, B-mode ultrasound provides valuable information about tumor morphology [9]. Despite these advantages, compared to MRI, the use of ultrasound for predictive modeling in BC remains underexplored. This gap is partly due to challenges inherent in ultrasound data, such as operator dependency and lower image consistency. Breast ultrasound is also characterized by a decreased specificity and positive predictive value [10]. Nonetheless, recent advances in deep-learning have shown the potential to overcome these limitations by identifying robust predictive features in imaging data [10,11,12,13]. Emerging three-dimensional (3D) B-mode ultrasound may further enhance lesion characterization through volumetric and more reproducible spatial information, reinforcing the potential of ultrasound as a tool for diagnostic assessment and predictive modeling [14].

Deep-learning, particularly convolutional neural networks (CNN), has become a key tool in medical image analysis. CNNs identify complex predictive patterns in imaging data, enabling applications from disease classification to treatment response prediction [15]. However, training CNNs for specialized tasks, such as NAC response prediction, requires large, annotated datasets, which are limited in medical imaging—especially ultrasound—due to privacy concerns, variability, and labor-intensive labeling processes [11]. Transfer learning and domain-specific pre-training can augment the training of models in the face of limited data. Transfer learning initializes models with weights from large datasets, exploiting the similarity in low-level features [16]. However, the distinct texture, resolution, and anatomical features in ultrasound limit the utility of transferring models from natural images [17,18,19]. Domain-specific pre-training offers an alternative to identify relevant features through more closely related tasks such as between malignancy classification and treatment response prediction [20,21,22]. This approach addresses data scarcity and improves performance, generalizability, and interpretability in ultrasound-based applications [18].

In this study, we used a deep-learning-based model to predict NAC response from pre-treatment B-mode ultrasound images. We compare two approaches employing a ResNet18 architecture [23]: (1) prediction using only imaging data and (2) integration of non-image clinical features in addition to imaging data. We evaluated three training approaches: training a model from random initialization (from scratch); transfer learning from ImageNet; and domain-specific pre-training of a model on publicly available ultrasound datasets. The comparison elucidates the role of pre-training in ultrasound-based predictive modeling. The evaluation assesses whether a deep-learning model using pre-treatment B-mode ultrasound images has the potential to predict response to NAC in BC patients, to stratify non-responders prior to NAC initiation. The main contributions of this work include demonstrating the potential of B-mode ultrasound as a predictive tool for the management of BC patients, and the improved performance achieved by using domain-specific pre-training in data-scarce environments. Furthermore, we explore the boundaries of multimodal data integration in this context, showing that the addition of clinical features benefits lower-performing models but reduces the accuracy of optimally trained image-only architectures.

## 2. Materials and Methods

### 2.1. Dataset

**Figure 1:**
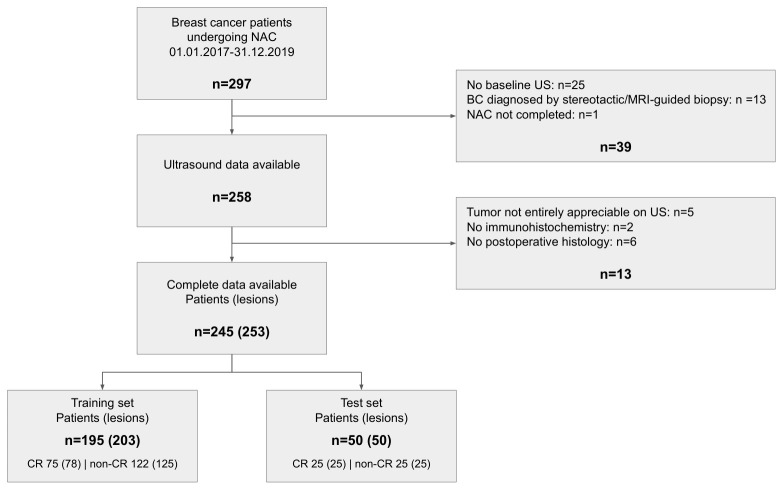
Flowchart of dataset selection.

This IRB-approved, retrospective study included data collected between 2017 and 2019. Due to its retrospective nature, the necessity for informed consent was waived by the IRB. We included all patients diagnosed with BC, for whom a baseline, pre-treatment ultrasound examination was available and who subsequently underwent a complete course of NAC at our hospital, a tertiary center. Patients were excluded if data on their treatment response could not be retrieved, if the entire lesion size could not be accurately measured sonographically, or if the maximum sonographic lesion diameter exceeded 10 cm. A total of 245 female patients with 253 lesions were included in the study (see Figure 1), with an average age of 55 years (range: 25–83 years); the clinical characteristics are given in Table 1. There were 198 examinations that had been performed at our hospital and 47 extramurally (either in smaller hospitals or in breast screening facilities). This patient cohort has been previously reported in [24]. Each lesion was classified regarding response to NAC based on the presence or absence of an invasive tumor in the results of the post-surgical pathology: if no invasive tumor was found in the breast or excised lymph nodes, the case was classified as CR. An in situ tumor component did not preclude CR [25]. Any case with residual invasive tumor in the breast or lymph nodes was classified as non-CR. Finally, 150 lesions (147 patients) were classified as non-complete response (non-CR), while 103 lesions (100 patients) were classified as complete response (CR).

The data were randomly divided into a training set of 203 lesions (195 patients) and a test set of 50 lesions (50 patients), with the test set balanced between 25 CR and 25 non-CR cases and unseen until the final evaluation. Only patients with a single lesion were included in the test set, to prevent information leakage. The training set consisted of 78 CR and 125 non-CR samples. The image dataset consisted of 24-bit RGB JPEG images, for which the physical scale and field of view were not standardized across samples. Due to the inherent operator dependence and variability in acquisition settings of ultrasound imaging—resulting in inconsistent spatial resolution, imaging depth, and field of view across subjects—preprocessing steps were applied, including cropping to remove border artifacts and the resizing of images to a standardized image size of 460×580, the approximate rounded average size over all samples. For domain-specific pretraining, we used the publicly available ultrasound datasets, BUSI [26] and Breast-Lesion-USG [27], which contain labels for malignant, benign, and normal cases.

**Table 1:** Clinical characteristics, retrieved from pre-treatment ultrasound examinations and pre-treatment ultrasound-guided biopsies. Molecular subtypes determined according to the 2013 St. Gallen consensus [28]. *p* values refer to the results of the univariate logistic regression. Abbreviations: ICNST = invasive carcinoma, no special type; DCIS = ductal carcinoma in situ; ILC = invasive lobular carcinoma.

Clinical Characteristic	CR	Non-CR	*p* Value
Total lesions	103	150	
Age (average)	25–83 (53)	29–80 (56)	0.1001
Tumor size (average)	5–60 (21) mm	7–100 (28) mm	0.0004
**Laterality**			0.9585
Right	52 (50%)	74 (49%)	
Left	51 (50%)	76 (51%)	
**Tumor grade**			0.0290
Tumor grade I (%)	1 (1%)	4 (3%)	
Tumor grade II (%)	13 (13%)	43 (28%)	
Tumor grade III (%)	89 (86%)	103 (69%)	
**Molecular subtype**			<0.0001
Luminal A (%)	0 (0%)	4 (2%)	
Luminal B HER2- (%)	14 (14%)	73 (49%)	
Luminal B HER2+ (%)	25 (24%)	27 (18%)	
HER2 positive (%)	22 (21%)	12 (8%)	
Triple-negative (%)	42 (41%)	34 (23%)	
**Histopathological type**			0.5400
ICNST no DCIS (%)	58 (56%)	93 (62%)	
ICNST DCIS (%)	39 (38%)	35 (23%)	
ILC (%)	2 (2%)	12 (8%)	
Other histopathological type (%)	4 (4%)	10 (7%)	

### 2.2. Non-Image Features

Through a search of the hospital information system, we recorded the clinicopathological features of each lesion. These were divided into tumor features (maximum tumor size, as measured on the baseline US images, tumor grade, molecular subtype and patient age), pathology (histo) features (estrogen and progesterone receptor as well as HER2 status), and BI-RADS [29] descriptors, independently evaluated by two breast imaging fellows as described in [24] (tissue composition, shape, orientation, margin, echo pattern, posterior features, presence of calcifications, architectural distortion, skin changes, duct changes, and presence of edema). A detailed description of the features is provided in Table 2.

### 2.3. Model Development

We developed a deep-learning model to predict the response to NAC in BC patients using pre-treatment B-mode ultrasound images. Our approach was based on a ResNet18 architecture, incorporating two distinct modeling strategies.

The first model used only the pre-treatment ultrasound images as input for the prediction model (Model: **Image**). Given a US image, the ResNet18 [23] encoder extracted deep feature representations. These representations were then passed through fully connected layers for classification. For the second approach, we evaluated the impact of incorporating different non-image feature sets via feature fusion (FF). The ultrasound images were processed by a ResNet18 encoder to obtain an image latent space feature vector. The non-image clinical and demographic data of the patient were incorporated to obtain the final representation by concatenating the image-derived features and non-image features. The final representations were then passed through fully connected layers for classification. Non-image features included either the **Tumor** features or the **Histo** features or the **BIRADS** descriptors of each tumor. In addition, we examined a **Combined** feature approach, which integrated all the aforementioned categories (Table 2).

### 2.4. Evaluation and Statistical Analysis

**Figure 2:**
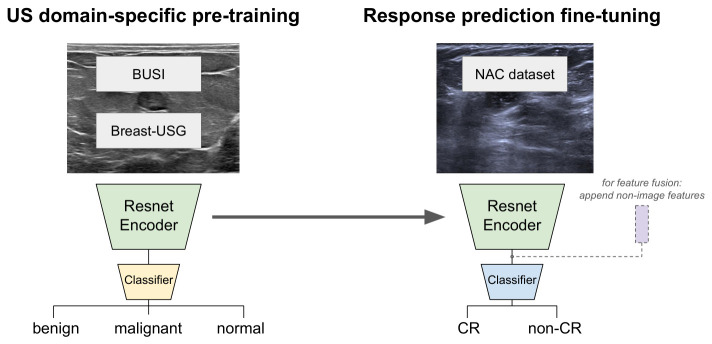
Overview of the US-domain-specific pre-training (USP) method. The classification network is first pre-trained to classify malignancy (benign, malignant, normal) using publicly available ultrasound datasets (BUSI [26] and Breast-Lesion-USG [27]). Then the pre-trained encoder is used as a feature extractor and fine-tuned for the NAC response prediction with a new classification head. Feature fusion is used for models including non-image features as indicated by dashed lines.

We evaluated three distinct training methods. (1) Training from scratch (SC): the ResNet18 weights were randomly initialized using He initialization [30]. (2) Transfer learning (TL): we set the model parameters to pre-trained weights from ImageNet, which were originally optimized for natural image classification, as the initial network state before supervised training. (3) US-domain-specific pre-training (USP): the network was first pre-trained to classify malignancy (normal, benign, malignant) using publicly available ultrasound datasets, specifically BUSI [26] and Breast-Lesion-USG [27], before being fine-tuned for the target task on the study data and labels (see Figure 2). Models were trained with cross-entropy loss and optimized using the Adam optimizer [31]. We employed data augmentation techniques, including random noise addition and flipping, to enhance generalization. Hyperparameters (type of augmentation, random chance, and number of training epochs) were optimized through five-fold cross-validation on the training set by observing training curves and model performance. Final performance was assessed through training on the complete training set and evaluation on the independent, previously unseen test set. Standard classification metrics (e.g., accuracy, ROC AUC) were calculated to evaluate model performance. Given the small test set (n=50), Bayesian bootstrap resampling [32,33] was used to estimate posterior distributions and Wilcoxon signed-rank tests were used to compare models [34]. The Mann–Whitney U test and the chi-squared test were used to compare continuous and categorical variables, as appropriate. Univariate logistic regression was used in the training set to identify statistically significant clinicopathological and imaging features.

To interpret model decisions, we applied Gradient-weighted Class Activation Mapping (Grad-CAM) [35] on test images to visualize class-specific saliency maps. Overlays on ultrasound images allowed assessment of whether model attention aligned with clinically relevant regions—an essential aspect in medical imaging.

All experiments were implemented in Python. Deep-learning models were developed using PyTorch (v1.8.1), while scikit-learn (v0.24.1) and SciPy (v1.5.4) were employed for evaluation metrics, statistical analyses, and related testing procedures. Model interpretability and gradient-based attribution analyses (Grad-CAM) were conducted using the Captum library (v0.4.1).

## 3. Results

Our study utilized a B-mode ultrasound dataset comprising 245 female BC patients (age 25–83, average 55), with 253 scans collected prior to treatment. Pathology-confirmed treatment response labels to NAC were categorized as CR (103 lesions, 100 patients) or non-CR (150 lesions, 147 patients). We evaluated the performance of three training strategies, SC (training from scratch), TL (transfer learning), and USP (US domain-specific pretraining), across five models: Image; Tumor; Histo; BIRADS; and Combined.

**Figure 3:**
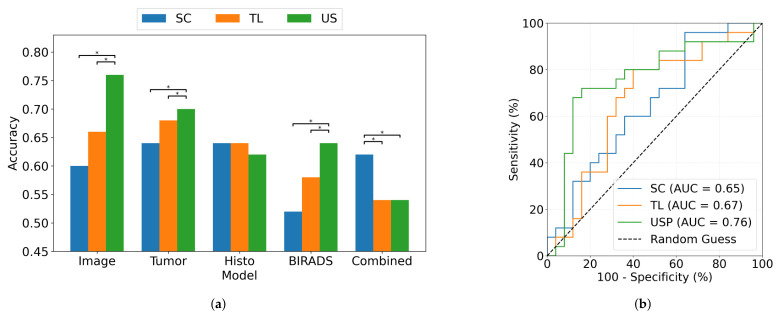
(**a**) Bar plot comparing the accuracies for different models (Image, Tumor, Histo, BIRADS, Combined). The models were trained using ultrasound images, incorporating three training setups: (1) training from scratch (SC); (2) transfer learning using ImageNet weights as initialization (TL); and (3) US domain-specific pre-training (USP), followed by fine-tuning for the response prediction task. (**b**) ROC curve of the Image model illustrating the performance of the deep-learning classification model to predict treatment response to neoadjuvant chemotherapy. Significant differences are highlighted with an * above horizontal bars in (**a**).

The prediction of NAC CR from pre-treatment US achieved 0.76 accuracy (specificity: 0.80, sensitivity: 0.72) for the USP Image model. As can be seen in Figure 3, the USP models achieved the highest classification accuracy for Image (0.76), Tumor (0.70), and BIRADS (0.64). These results were significantly (*p* < 0.05) better than those obtained with both SC (Image: 0.60, Tumor: 0.64, BIRADS: 0.52) and TL (Image: 0.66, Tumor: 0.68, BIRADS: 0.58). TL also outperformed SC significantly (*p* < 0.05) with these features. In contrast, the best performance for the Combined features was achieved using SC (0.62), which significantly (*p* < 0.05) surpassed both TL (0.54) and USP (0.54). For the Histo features, all three training methods yielded comparable accuracies (SC: 0.64, TL: 0.64, USP: 0.62), with no statistically significant differences observed among them. Specificity, sensitivity, accuracy and ROC AUC for each model and training method are summarized in Table 3. Regarding misclassified cases, the USP Image model erroneously predicted seven cases as achieving CR and five cases as non-CR.

**Figure 4:**
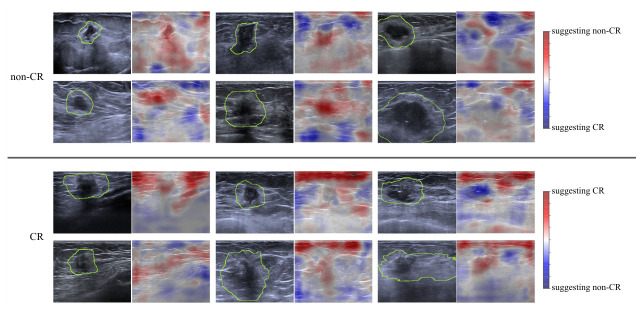
Grad-CAM visualizations for treatment response prediction from breast ultrasound images. The rows show six representative examples from patients predicted as non-complete responders (non-CR) and complete responders (CR). For each example, the original ultrasound scan is displayed, with highlighted tumor and peritumoral position, as well as with a Grad-CAM heatmap overlay, highlighting image regions that contributed most to the model’s prediction. The heatmap indicates that, for non-CR cases, the model focused on the tumor and the retrotumoral posterior shadowing, whereas, for CR cases, it emphasized more superficial peritumoral regions with less attention to the tumor itself.

**Figure 5:**
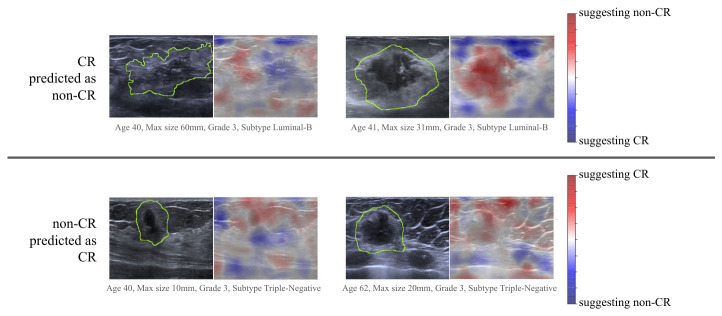
Grad-CAM visualizations for incorrect treatment response predictions from breast ultrasound images. The rows show two representative samples from patients incorrectly predicted as non-complete responders (non-CR) and complete responders (CR). For each example, the original ultrasound scan is displayed, with highlighted tumor and peritumoral position, as well as with a Grad-CAM heatmap overlay, highlighting image regions that contributed most to the model’s prediction. Tumors incorrectly predicted as non-CR were often larger and more heterogeneous, suggesting the model relied on features typically associated with poorer response that are not universally predictive. Conversely, tumors incorrectly predicted as CR tended to be more homogeneous and hypoechoic with superficial activation, indicating the model also captures broader contextual and clinically plausible patterns that can mislead predictions.

The Grad-CAM [35] saliency maps were overlaid on the original images to facilitate visual interpretation. Six representative examples from each target class (CR and non-CR) were selected and are presented in Figure 4. Misclassified examples are shown in Figure 5. In the non-CR cases, the Grad-CAM heatmaps consistently highlighted regions that corresponded to the tumor mass, as well as to the area directly posterior to the tumor. This retro-tumoral region is often associated with acoustic shadowing, a feature frequently observed in luminal-type breast tumors [36,37]. In contrast, the Grad-CAM maps for the CR cases emphasized different spatial patterns. In addition to the tumor region, the model focused on more superficial areas closer to the skin surface, the irregular contours of the tumor, and the regions surrounding the tumor. These distinctions suggest that the model exploits different visual cues depending on the predicted treatment response.

## 4. Discussion

In this study, we addressed the critical challenge of predicting NAC response in BC patients using pre-treatment B-mode ultrasound images. We developed a deep-learning ResNet18-based model and compared two modeling approaches: “Image,” which relied solely on imaging data, and “FF,” which integrated non-image clinical features. We evaluated three training strategies—training from scratch (SC), transfer learning (TL), and US-domain-specific pretraining (USP)—across “Image” and four FF model configurations, the latter using Tumor features, Histopathological (Histo) features, BIRADS descriptors, and Combined features. The results provide important insights into both the utility of auxiliary information and the effectiveness of training strategies in this clinical prediction task.

The USP Image model achieved the highest overall accuracy (0.76), outperforming all other models, including those that incorporated additional clinical features. This suggests that raw ultrasound images contain rich, predictive information about NAC response—and that USP is particularly effective at extracting it.

**Domain-specific pretraining improves performance, especially for image-based models.** USP significantly outperformed both SC and TL, achieving the best results in the Image (0.76), Tumor (0.70), and BIRADS (0.64) models. These improvements highlight the value of pretraining on large amounts of US data, enabling models to better capture morphology and texture patterns specific to breast ultrasound, which are not well represented in natural image datasets.

**The contribution of non-image features was varied with regard to model performance.** Tumor and Histo models trained with SC or TL outperformed the corresponding Image versions, showing that non-image features can add complementary information when the image encoder is less capable. However, with USP, the benefit of adding clinical features diminished—and, surprisingly, the Combined model performed worse with USP and TL (both 0.54) than with SC (0.62). This suggests that USP enables the model to extract much of the relevant information directly from the image, making additional features redundant—or even detrimental if they introduce conflicting signals. We did not observe any systematic bias related to the clinical variables during model training or evaluation, so one possible explanation is that USP-trained encoders learn feature hierarchies optimized for imaging data alone and struggle to integrate heterogeneous inputs effectively. In contrast, SC models learn all features jointly from the start, potentially allowing better synergy across modalities. These results underline the importance of careful multimodal integration strategies, especially when using domain-specific pretrained encoders, as was the case in our USP models.

Understanding how an AI algorithm makes classification decisions is essential to increase clinicians’ trust in AI systems. One way to achieve this is through the use of heatmaps. The Grad-CAM analysis used in our study provides important insight into the decision-making process of the deep-learning model and highlights the distinct image regions associated with different treatment outcomes. In non-responding cases, the model primarily attended to the tumor and the retro-tumoral acoustic shadow. This shadowing is a known sonographic feature often linked to dense, fibrotic tumors, particularly those of the luminal subtypes, which are less likely to respond completely to NAC [37]. The model’s focus in this area suggests it may have learned to associate this imaging phenotype with chemoresistance. Conversely, in cases predicted to achieve a complete response, the importance maps extended beyond the tumor itself. The highlighted regions included more superficial areas, as well as peritumoral tissue, with no abrupt interface toward the tumor itself, which corresponded to indistinct tumor margins and even areas of posterior enhancement—potential indicators of more aggressive, yet chemo-sensitive tumor subtypes, such as triple-negative or HER2-positive cancers [37]. The attention to tumor surroundings may also reflect subtle peritumoral changes, such as edema or early inflammatory response, which are not readily assessed in standard clinical interpretation but may carry prognostic information, as has been previously demonstrated [38]. An analysis of misclassified cases provided even more insight into the model’s decision-making, highlighting not only learned patterns but also current limitations. Among tumors that achieved complete response but were predicted as non-CR, lesions were frequently larger and exhibited a heterogeneous echotexture—features that are typically associated with poorer treatment response. This suggests that, in these instances, the model may have relied on imaging characteristics that are generally predictive but not universally applicable. Conversely, in tumors that did not achieve CR but were predicted as CR, Grad-CAM maps often demonstrated pronounced activation in superficial regions, particularly the skin, a finding that remains difficult to interpret conclusively. This observation may indicate that the model, in some cases, may have incorporated broader contextual image features rather than focusing exclusively on the tumor itself. In addition, these tumors were often relatively homogeneous and markedly hypoechoic. Such imaging characteristics are commonly observed in triple-negative breast cancers, which are known to exhibit higher response rates to therapy. This suggests that the model has learned meaningful associations between certain visual features and treatment response, even though these associations may not hold in all cases. Overall, while misclassifications underscore the current limitations of the model, they also reveal that its predictions are frequently grounded in clinically plausible imaging patterns, reflecting a degree of learned, interpretable behavior. These observations underscore the potential of deep-learning models not only for predictive tasks, but also for uncovering imaging biomarkers.

Previous studies on NAC response prediction in BC primarily used QUS features with SVM or KNN classifiers on small datasets (e.g., 56–96 patients), reporting accuracies of 78–88% [39,40,41]. CEUS-based logistic regression has also been explored, showing that, from all pre-treatment CEUS features, only “internal homogeneity” was a significant, independent predictor of CR in multivariate analysis, with an AUC of 0.71 [42]. One recent study used pre-treatment B-mode ultrasound, achieving an AUC of 0.72 with logistic regression on visually identified features [24]. Our deep-learning model outperformed this approach across all metrics, demonstrating the potential of implementing a deep-learning technique. Unlike prior work that relied on specialized modalities or expert annotations, our method does not need human annotation, and uses standard B-mode ultrasound without contrast agents or custom hardware. Although the performance improvement may appear marginal compared to [24], our approach, based only on 2D, B-mode US images, also addresses the user dependence of subjective BI-RADS descriptor evaluations, as well as the possible lack or inadequacy of clinicopathological features in resource-restricted clinical settings, thus offering improved and more robust performance, broader accessibility, and better scalability.

MRI is widely used to predict NAC response [43], with reported accuracies ranging from 74% in large cohorts [7] to 82–91% using deep-learning on DCE-MRI [8,44,45]. Our model achieved 76% accuracy, within this range but on the lower end of deep-learning-based MRI methods. While MRI appears to offer higher predictive accuracy due to superior imaging capabilities, ultrasound is more accessible and cost-effective. Improving ultrasound-based models is, therefore, crucial for scalable, real-world impact.

Despite these promising results, several limitations must be acknowledged. First, our analysis was conducted on a single dataset with a relatively small sample size, which may limit the generalizability of our findings. However, this dataset is uniquely annotated with treatment response labels, providing an important foundation for future research in this area. Second, while we employed rigorous data-splitting techniques, including training, validation, and testing subsets, the absence of an external validation cohort precludes a full evaluation of the model’s performance on entirely unseen data. However, our cohort consisted of almost 20% extramural images, acquired with devices from various vendors. Therefore, our model was both trained and tested in diverse cohorts, representative of the workload of a tertiary center. That said, as soon as an independent dataset becomes available, our methodology can be readily applied for external validation, making this an easily addressable limitation. Finally, our study exclusively used B-mode ultrasound. It is likely that incorporating additional functional imaging techniques (e.g., elastography, Doppler or CEUS) could further improve predictive performance. Yet, B-mode is the most widely used ultrasound modality, enhancing the generalizability of our findings and ensuring broad clinical applicability.

## 5. Conclusions

This study underscores the untapped potential of ultrasound in predictive oncology, particularly in resource-constrained settings where MRI is not readily available. By integrating domain-specific pre-training and clinical features, we have demonstrated that deep-learning models can achieve meaningful performance using pre-treatment ultrasound data to predict NAC response in BC patients, making this modality a viable and accessible alternative. Our findings establish the groundwork for future research to expand the use of ultrasound in BC treatment and illustrate the potential of combining advanced imaging technologies with artificial intelligence for personalized medicine.

## Figures and Tables

**Table 2:** Overview of the non-image features and their feature set correspondence. Histo data, molecular subtype, and tumor grade were acquired from pathology reports of the pre-treatment, ultrasound-guided biopsy. Tumor size and BI-RADS descriptors were based on pre-treatment ultrasound images only. For more details on histopathological data of the patient cohort, see [24].

Feature Set	Feature
**Tumor**	Max size (mm/100)
	Molecular subtype (Luminal, HER2+, TNBC)
	Grade (low, high)
	Age (years/100)
**Histo**	ER status (negative, positive)
	PR status (negative, positive)
	HER2 status (negative, positive)
**BIRADS**	Tissue composition (homogenous fatty, homogeneous fibroglandular, heterogeneous)
	Shape (oval, round, irregular)
	Margin (circumscribed, indistinct, microlobulated, angular, spiculated)
	Orientation (parallel, non-parallel)
	Echo pattern (anechoic, hyperechoic, isoechoic, hypoechoic,
	complex cystic and solid, heterogeneous)
	Posterior features (none, enhancement, shadowing, combined)
	Calcifications (no, yes)
	Edema (no, yes)
	Skin changes (no, yes)
	Duct changes (no, yes)

**Table 3:** Evaluation metrics (Specificity, Sensitivity, Accuracy and ROC AUC) for each model (Image, Tumor, Histo, BIRADS, and Combined) trained with three different strategies: SC (training from scratch); TL (transfer learning from ImageNet); and USP (US-domain-specific pre-training). Bold font indicates the best values for each approach; (*) highlights a significantly better value for ROC AUC (*p* < 0.05); 95% confidence intervals are shown in brackets.

		Metric			
Training Approach	Model	Specificity	Sensitivity	Accuracy	ROC AUC
**Training** **from scratch** **(SC)**	Image	0.60 [0.40, 0.77]	0.60 [0.40, 0.78]	0.60 [0.46, 0.73]	0.65 [0.49, 0.78]
	Tumor	**0.68** [0.50, 0.84]	0.60 [0.42, 0.77]	**0.64** [0.51, 0.76]	0.66 [0.50, 0.80]
	Histo	0.44 [0.28, 0.67]	**0.84** [0.46, 0.81]	**0.64** [0.42, 0.70]	0.58 [0.41, 0.74]
	BIRADS	0.48 [0.29, 0.67]	0.56 [0.37, 0.74]	0.52 [0.38, 0.66]	0.62 [0.46, 0.77]
	Combined	0.64 [0.45, 0.82]	0.60 [0.41, 0.78]	0.62 [0.49, 0.75]	**0.68** * [0.54, 0.81]
**Training** **learning** **(TL)**	Image	0.64 [0.45, 0.81]	**0.68** [0.49, 0.85]	0.66 [0.53, 0.78]	0.67 [0.50, 0.81]
	Tumor	**0.80** [0.64, 0.93]	0.56 [0.39, 0.75]	**0.68** [0.55, 0.80]	**0.71** * [0.56, 0.84]
	Histo	0.68 [0.49, 0.85]	0.60 [0.41, 0.79]	0.64 [0.50, 0.77]	0.67 [0.52, 0.81]
	BIRADS	0.68 [0.50, 0.84]	0.48 [0.29, 0.69]	0.58 [0.45, 0.72]	0.64 [0.49, 0.79]
	Combined	0.52 [0.32, 0.71]	0.56 [0.37, 0.75]	0.54 [0.40, 0.67]	0.59 [0.42, 0.73]
**Domain-specific** **pre-training** **(USP)**	Image	**0.80** [0.62, 0.93]	**0.72** [0.53, 0.88]	**0.76** [0.63, 0.87]	**0.76** * [0.59, 0.89]
	Tumor	0.72 [0.53, 0.88]	0.68 [0.50, 0.83]	0.70 [0.57, 0.81]	0.71 [0.55, 0.84]
	Histo	0.68 [0.48, 0.85]	0.56 [0.36, 0.74]	0.62 [0.48, 0.75]	0.64 [0.47, 0.78]
	BIRADS	0.64 [0.44, 0.81]	0.64 [0.44, 0.80]	0.64 [0.52, 0.76]	0.67 [0.50, 0.80]
	Combined	0.44 [0.26, 0.65]	0.64 [0.46, 0.82]	0.54 [0.41, 0.67]	0.50 [0.35, 0.66]

## Data Availability

The raw data supporting the conclusions of this article will be made available by the authors on request.

## Abbreviations

The following abbreviations are used in this manuscript:

NAC	Neoadjuvant chemotherapy
CR	Complete response
SC	Training from scratch
TL	Transfer learning
USP	Ultrasound domain-specific pre-training
CAM	Class activation map
BC	Breast cancer
MRO	Magnetic resonance imaging
US	Ultrasound
CNN	Convolutional neural network
TNBC	Triple-negative breast cancer
FF	Feature fusion
ICNST	Invasive carcinoma, no special type
DCIS	Ductal carcinoma in situ
ILC	Invasive lobular carcinoma
